# Integrating omic approaches for abiotic stress tolerance in soybean

**DOI:** 10.3389/fpls.2014.00244

**Published:** 2014-06-03

**Authors:** Rupesh Deshmukh, Humira Sonah, Gunvant Patil, Wei Chen, Silvas Prince, Raymond Mutava, Tri Vuong, Babu Valliyodan, Henry T. Nguyen

**Affiliations:** National Center for Soybean Biotechnology and Division of Plant Sciences, University of MissouriColumbia, MO, USA

**Keywords:** abiotic stress tolerance, soybean, genomics, proteomics, transcriptomics, ionomics, phenomics

## Abstract

Soybean production is greatly influenced by abiotic stresses imposed by environmental factors such as drought, water submergence, salt, and heavy metals. A thorough understanding of plant response to abiotic stress at the molecular level is a prerequisite for its effective management. The molecular mechanism of stress tolerance is complex and requires information at the omic level to understand it effectively. In this regard, enormous progress has been made in the omics field in the areas of genomics, transcriptomics, and proteomics. The emerging field of ionomics is also being employed for investigating abiotic stress tolerance in soybean. Omic approaches generate a huge amount of data, and adequate advancements in computational tools have been achieved for effective analysis. However, the integration of omic-scale information to address complex genetics and physiological questions is still a challenge. In this review, we have described advances in omic tools in the view of conventional and modern approaches being used to dissect abiotic stress tolerance in soybean. Emphasis was given to approaches such as quantitative trait loci (QTL) mapping, genome-wide association studies (GWAS), and genomic selection (GS). Comparative genomics and candidate gene approaches are also discussed considering identification of potential genomic loci, genes, and biochemical pathways involved in stress tolerance mechanism in soybean. This review also provides a comprehensive catalog of available online omic resources for soybean and its effective utilization. We have also addressed the significance of phenomics in the integrated approaches and recognized high-throughput multi-dimensional phenotyping as a major limiting factor for the improvement of abiotic stress tolerance in soybean.

## Introduction

Soybean is the most important legume crop which provides sources of oil and protein for human as well as for livestock. Soybean also enhances soil fertility because of the symbiotic nitrogen fixing ability. Soybean contributed to more than 50% of globally consumed edible oil (SoyStats, 2013^1^). Apart from the consumption, soybean oil is being considered as a future source of fuel and efforts are being made to improve soy-diesel production (Candeia et al., 2009). Soybean protein-based bio-degradable materials are also being considered as an alternative for plastics (Song et al., 2011). Soybean products are gaining attention because of its pharmaceutical attributes such as anti-cancerous properties (Ko et al., 2013). Such diverse uses of soybean make it a more widely desired crop plant and are rapidly increasing its demand. In this regard, soybean yield improvement has been achieved by 1.3% per year (Ray et al., 2013). However, the increasing global population will need double the current food production by the year 2050 and at the current rate it can achieve only ~55% (Ray et al., 2013). It may be more difficult to produce sufficient yield with the changing climate. Therefore soybean yield prediction must consider the ongoing challenges of extreme weather such as drought, flood, heat, cold, frost, and possible UV stress.

Abiotic stresses are the most challenging of all major constraints in crop production. Soybean production is not only influenced by environmental factors, such as drought, water submergence, salt, and heavy metals, but it also faces challenges to get adapted in non-traditional areas. This demands extensive breeding for the development of local cultivars (Tanksley and Nelson, 1996; Grainger and Rajcan, 2013). Direct selection for yield stability based on multi-location trials has been traditionally used for the development of varieties adapted to adverse environmental conditions. This approach is more difficult for abiotic stress related traits because of low heritability and highly influenced by environmental conditions (Manavalan et al., 2009). Direct selection is also a time-consuming and labor intensive process. Strategic marker-assisted breeding can efficiently accelerate the development of tolerant cultivars; however, it also necessitates knowledge about genomic loci governing the traits and the availability of tightly linked molecular markers (Xu et al., 2012). Molecular marker development has been accelerated with the availability of sequenced genomes and organelles in crop plants (Singh et al., 2010; Sonah et al., 2011a; Tomar et al., 2014).

Marker-assisted breeding has become sophisticated with the availability of complete soybean genome sequence due to subsequent development of locus-specific molecular markers (Schmutz et al., 2010; Song et al., 2010). Genome-wide high density markers availability also facilitates the haplotype analysis and identification of different alleles for agronomical important traits (Tardivel et al., 2014). Marker-assisted breeding has been carried-out mostly for simple traits governed by a single, or at most a few loci (Shi et al., 2009; Jun et al., 2012). Marker-assisted breeding also suffers due to undesired genetic drag (Tanksley and Nelson, 1996; Shi et al., 2009). The genetic background of the recurrent parent also plays an important role in the phenotypic expression of newly introgressed gene(s) mostly because of the complex epistatic interaction (Palloix et al., 2009). In the case of multiple complex traits, epistatic interaction is more unpredictable and it is hard to develop a strategic breeding plan until unless solid information is available about the molecular mechanisms involved in the trait development. Recent technological development in genomics provides tremendous power to predict genetic factors, their evolution, distribution, and interactions at great extent (Morrell et al., 2011; Sonah et al., 2011b). Genetic engineering is the most advanced approach that has been used for the genetic improvement of soybean. Genetically modified (GM) soybean crops for insect-resistance and herbicide-tolerance has covered most of the cultivated area in the world (Carpenter, 2010). Although, GM soybean has proven to be very successful, it raises ethical controversies, and it is available only for few traits (Carpenter, 2010). Integration of multi-disciplinary knowledge is required to design future soybean varieties with ideal plant types providing high and stable yield in adverse climatic conditions. In this context, a detailed review was made to evaluate progress achieved in different omic approaches and to highlight future perspectives for its effective exploration toward the development of abiotic stress tolerant soybean cultivars.

## Omics approaches in the technological era

**Figure 1:**
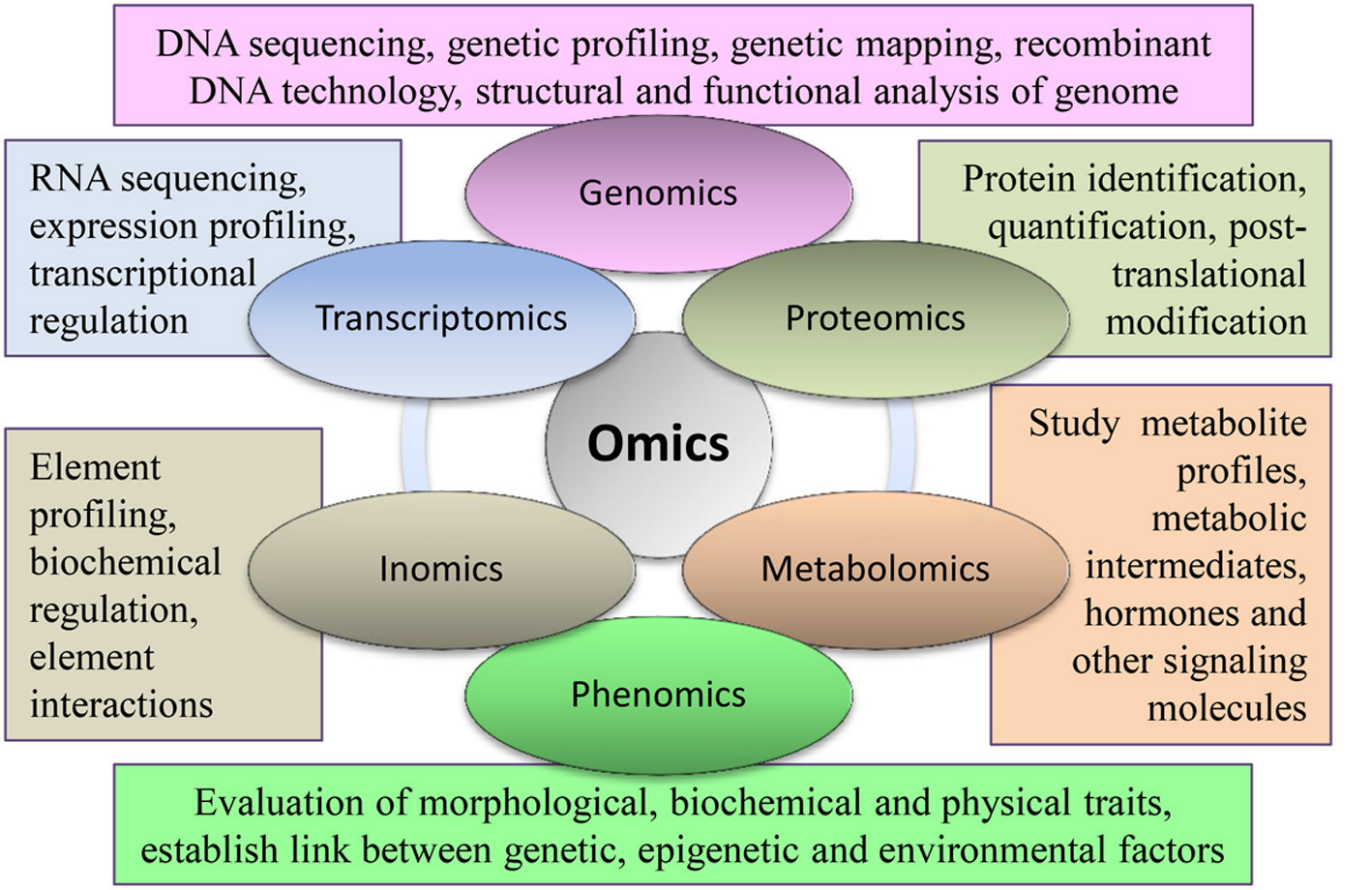
**Important branches of omics with their major components being used in different integrated approaches in soybean**.

Plant molecular biology aims to study cellular processes, their genetic control, and interactions with environmental changes. Such a multi-dimensional and detailed investigation requires large-scale experiments involving entire genetic, structural, or functional components. These large scale studies are called “omics.” Major components of omics include genomics, transcriptomics, proteomics, and metabolomics (Figure 1). These omics approaches are routinely used in various research disciplines of crop plants, including soybean. Omics approaches have improved very rapidly during the last decade as technology advances. Subsequently, high-throughput data developed by omic experiments require extensive computational resources for storage and analysis. Thus, several online databases, analysis servers, and omics platforms have been developed. Omics is getting broader coverage and it is anticipated that several new omic fields will evolve in near future.

## Genomics advances for abiotic stress tolerance in soybean

### Molecular marker resources

Genomic applications in soybean have become more standard with the availability of whole genome sequence (WGS) (Schmutz et al., 2010). The WGS provided the basis for the development of thousands of simple sequence repeat (SSR) markers and millions of single nucleotide polymorphism (SNP) markers (Song et al., 2010; Sonah et al., 2013). Recent developments in next generation sequencing (NGS) technologies make sequencing-based genotyping cost effective and efficient. Three main complexity reduction methods, namely Reduced Representation Libraries (RRLs), Restriction site Associated DNA (RAD) sequencing, and Genotyping-by-Sequencing (GBS) are being routinely used. Among these, GBS is gaining more attention because of its simplified and cost effective methodology (Elshire et al., 2011; Sonah et al., 2012). The GBS approach has been successfully used in several crop species (Poland and Rife, 2012). Recently, GBS methodology has been improved and streamlined for soybean (Sonah et al., 2013). However, sequencing-based genotyping methods require computational expertise and significant time for data analysis. This restricts its use in marker-assisted breeding where timely selection is very important. GBS will be widely used in the future with an increasing number of software packages and computational pipelines (Sonah et al., 2013).

Technological advances have also provided a high-throughput, reliable, and quick array-based genotyping platforms. The SNP array development require initial information about SNPs, fortunately, information about millions of SNPs is already available in the public domain (Table 1). The Illumina Infinium array (SoySNP50K iSelect BeadChip) for ~50,000 SNPs has been successfully developed and used for the genotyping of several soybean plant introduction (PI) lines (Song et al., 2013). Technological advances beyond this make it possible to re-sequence hundreds of lines in a cost effective manner and has started a new era of genotyping by re-sequencing (Lam et al., 2010; Li et al., 2013; Xu et al., 2013). Now, the challenge for plant biologists is how to effectively use these resources for marker-assisted applications.

**Table 1:** **List of significant studies performed to develop SNP markers and subsequent genotyping using different technological platforms in soybean**.

**Sr. No**	**Genotyping platform/Approach**	**Genotypes**	**SNPs**	**References**
1	Illumina GoldenGate assay	3 RIL mapping populations	384	Hyten et al., 2008
2	Illumina Infinium SoySNP6K BeadChip	92 RILs	5376	Akond et al., 2013
3	Illumina genome analyzer/Reduced Representation Libraries (RRLs)	5 diverse genotypes	14,550	Varala et al., 2011
4	Illumina GoldenGate assay	3 RIL mapping populations	1536	Hyten et al., 2010b; Vuong et al., 2010
5	Illumina genome analyzer /RRLs	444 RILs	25,047	Hyten et al., 2010a
6	Illumina GAIIx/Genotyping by sequencing (GBS)	8 diverse genotypes	10,120	Sonah et al., 2013
7	Illumina Genome Analyzer II/whole genome re-sequencing	17 wild and 14 cultivated	2,05,614	Lam et al., 2010
8	Illumina Genome Analyzer II/whole genome re-sequencing	25 diverse genotypes	51,02,244	Li et al., 2013
9	Illumina genome analyzer/RRLs	Parental lines of mapping population	39,022	Wu et al., 2010
10	Illumina Infinium BeadChip	96 each of landraces, elite cultivars and wild accessions	52,041	Song et al., 2013

### QTL mapping for abiotic stress tolerance in soybean

Genetic fingerprinting, linkage mapping, and quantitative trait loci (QTL) mapping are marker based applications that have become more sophisticated with the availability of different genotyping platforms (Table 1). Consequently, several efforts have been made to identify QTL for abiotic stress tolerance in soybean (Table S1). QTL studies have identified thousands of QTL spanning the entire genome (www.soykb.org, www.soybase.org). This is due to the complex inheritance of abiotic stress tolerance which has identified unstable QTL across different environments. Further utilization of QTL information for marker-assisted breeding or candidate gene identification has become difficult due to this complexity. Statistical tools such as “Meta-QTL analysis” have been advanced that compile QTL data from different studies together on the same linkage map for identification of precise QTL region (Deshmukh et al., 2012; Sosnowski et al., 2012). Several efforts have been performed to identify meta-QTL for different agronomical and quantitative traits in soybean (Table 2). Meta-analysis studies are still required exclusively for abiotic traits.

**Table 2:** **Meta-QTL studies performed for different traits in soybean**.

**Sr. No**	**Trait**	**Meta QTL**	**QTL compiled**	**Studies compiled**	**References**
1	Soybean cyst nematode resistance	7	62	17	Guo et al., 2006
2	Soybean cyst nematode resistance	16	151	19	Zhang et al., 2010
3	Seed oil content	20	121	22	Qi et al., 2011b
4	Seed oil content	25	130	39	Qi et al., 2011a
5	100-seed weight	17	65	12	Zhao-Ming et al., 2009
6	100-seed weight	15	117	13	Sun et al., 2012a
7	Fungal disease resistance	23	107	23	Wang et al., 2010
8	Insect resistance	20	81	–	Jing et al., 2009
9	Seed protein content	23	107	29	Zhao-Ming et al., 2011
10	Plant height	12	93	13	Sun et al., 2012b
11	Phosphorus efficiency	29	96	–	Huang et al., 2011
12	Growth stages	9	98	10	Qiong et al., 2009

### Genome-wide association studies (GWAS) in soybean

QTL mapping using bi-parental populations has limitations because of restricted allelic diversity and genomic resolution. The allelic diversity can be increased to some extent by using multi-parental crosses. Recently, Multi-parent Advanced Generation Inter-Cross populations (MAGIC) has been used to identify QTL for blast and bacterial blight resistance, salinity and submergence tolerance, and grain quality traits in rice (Bandillo et al., 2013). Such multi-parental populations has mapping resolution limitations since it depends on meiotic events (crossing-over) (Kover et al., 2009). In contrast, the genome-wide association study (GWAS) approach provides opportunities to explore the tremendous allelic diversity existing in natural soybean germplasm. Mapping resolution of GWAS is also higher since millions of crossing events have been accumulated in the germplasm during evolution.

GWAS is routinely being used in many plant species, but only a few studies have been reported in soybean (Table S2). These studies were performed with limited markers and genotypes. GWAS in soybean is lagging behind compared to maize, mostly because of the slow linkage disequilibrium (LD) decay (Hyten et al., 2007; Mamidi et al., 2011). Another serious problem is the confounding population structure since it may cause spurious associations leading to an increased false-discovery rate (FDR). Studies that involve case-control phenotypes (binary) carefully relate the cases and controls to minimize confounding effects. GWAS for quantitative traits like abiotic stress tolerance are predictable to be affected by a confounding population. Different models have been developed for population stratification and spurious allelic associations like MLM and CMLM which takes into account the population structure and kinship. Recently, GWAS for *Sclerotinia sclerotiorum* resistance was performed using 7864 SNPs in soybean (Bastien et al., 2014). The study provided details of a probable marker requirement and methodologies involving population stratification for effective GWAS (Bastien et al., 2014). Development in statistical tools, genotyping methods, and studies involving larger sets of genotypes will definitely improve GWAS power in soybean.

### Genomic selection (GS) in soybean

Marker-assisted breeding for simple Mendelian traits are easy and effective, but it can be problematic for the complex traits such as abiotic stresses that are generally polygenic. Even major QTLs can explain only a small fraction of phenotypic variation and may show unexpected trait expression in new genetic backgrounds because of epistatic interactions. These limitations can be effectively addressed by the use of an approach called “Genomic-selection” (GS). GS is relatively simple, more reliable, and a more powerful approach where breeding values of lines are predicted using their phenotypes and marker genotypes (Heffner et al., 2009). GS is more effective since it uses all marker information simultaneously to develop a prediction model avoiding biased marker effects (Heffner et al., 2009). GS captures small-effect QTL that governs most of the variation including epistatic interaction effects.

An overview of research articles regarding GS published during last decade showed exponential growth within recent years (Figure S1). The increasing popularity of GS among plant as well as animal breeders is mostly because of the reduced cost of genotyping. Currently, GS is being used for breeding in several different crops (Table S3). In soybean, efforts have been made to evaluate GS using different models. A GS study in soybean has used 126 recombinant inbred lines and 80 SSR markers to predict primary embryogenesis capacity which is a highly polygenic trait (Hu et al., 2011). In this report, high correlation (*r*^2^ = 0.78) has been observed among the genomic estimated breeding value (GEBV) and the phenotypic value. Another study published recently using 288 cultivars and 79 SSR markers, found a correlation coefficient of 0.90 among the GEBV and the phenotypic value (Shu et al., 2012). Both the reports have shown high accuracy of prediction but only with a few markers and genotypes. Predicting the accuracy of GS will need more investigations involving high-throughput genotyping of larger populations evaluated across different environments.

Accuracy of GS largely depends on genetic × environmental (G × E) interaction but most of the studies focused only on an estimation of the main effect for each marker. These multi-environmental trials are of prime importance for plant breeding not only to study G × E but especially to increase the number of breeding cycles per year. The challenge for GS is to get accurate GEBV in respect to the G × E effect. Considering environmental effects is not new for plant breeders and most statistical models used for multi-location trials do reflect G × E (Hammer et al., 2006). It is also more common in QTL mapping studies where QTL × environment interaction evaluations were utilized to estimate QTL effect.

Improved factorial regression models have been proposed recently for GS that consider stress covariates derived from daily weather data (Heslot et al., 2014). This model has shown increased accuracy by 11.1% for predicting GEBV in unobserved environments where weather data is available (Heslot et al., 2014). This study suggests possible utilization of phenotypic data and historical data of weather conditions accumulated over decades in different soybean breeding programs. Similar information can be used for abiotic stress tolerance improvement in soybean.

### Combining marker-assisted breeding with genomic selection

**Figure 2:**
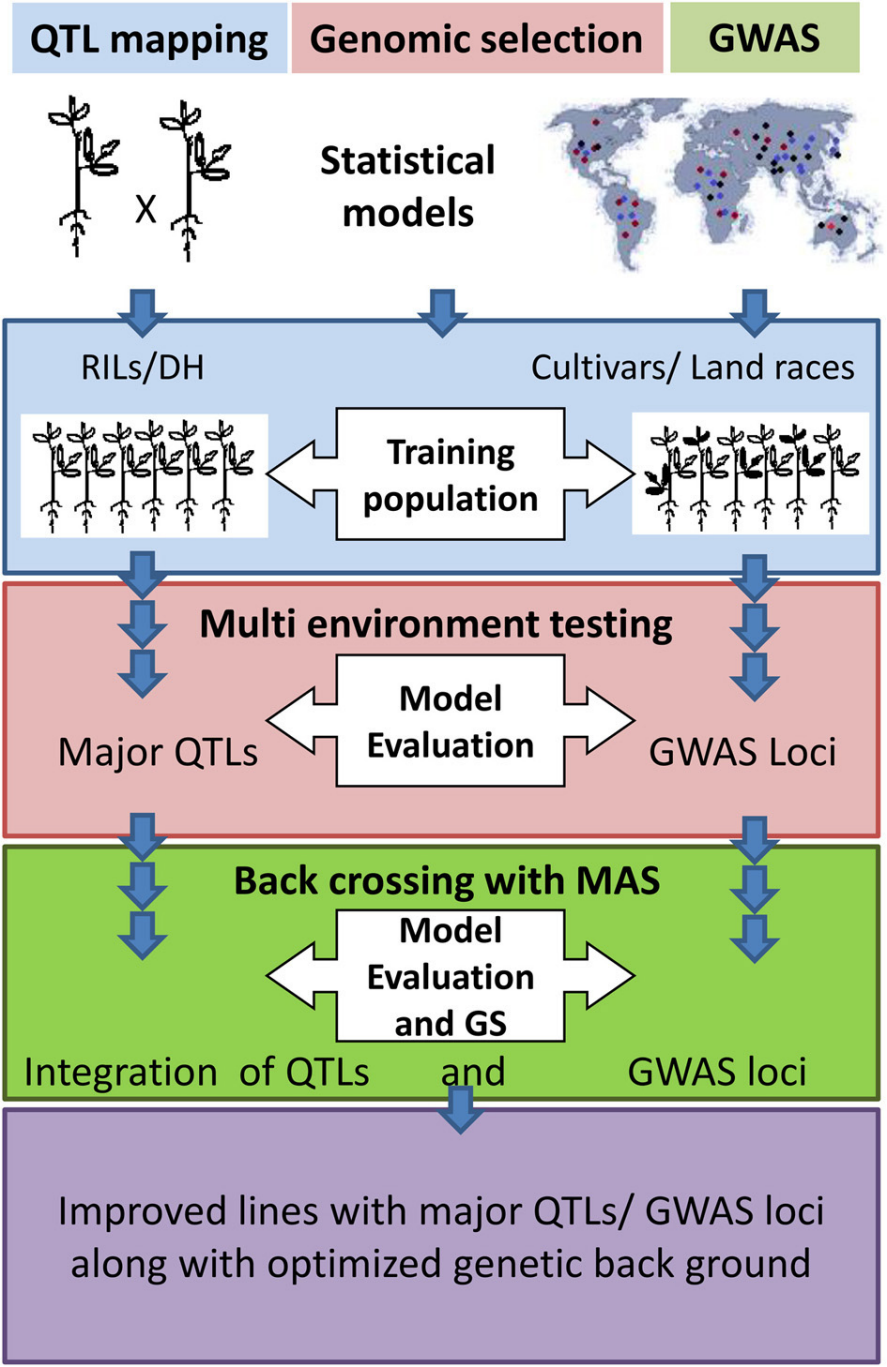
**Combined approach of QTL mapping/Genome-wide association study (GWAS) and Genomic selection (GS)**.

Molecular marker genotyping is a common requirement for QTL mapping, GWAS, and GS and can be the basis for combining these approaches (Figure 2). Most of the GS studies have used recombinant inbred line (RIL) populations to train the prediction model (Table S3). Therefore, GS and QTL mapping can be performed simultaneously. A set of diverse cultivars can be used for GWAS and GS all together (Table S3). In the marker-assisted breeding, introgression of QTL or GWAS loci to well adapted cultivar is performed. The donor line (for QTL or GWAS loci) may be wild or low yielding line. Therefore, several cycles of backcrossing are performed to retain the genetic background of the recipient parent (the adapted cultivar) except for the QTL/GWAS loci which represent the donor background. Nevertheless, GS does not provide control over the genetic background and this may be problematic when the donor is not an adapted line. In addition, GS cannot guarantee for major QTL which are already known. Therefore, information about QTL/GWAS loci should be incorporated with GS models so that the balance of genetic background can be made along with maximum gain of breeding value.

## Transcriptome profiling for abiotic stress tolerance

Plants, including soybean, responses to external environments is very complex. A wide range of defense mechanisms are activated that increases plant tolerance against adverse conditions in order to avoid damage imposed by abiotic stresses. The first step toward stress response is stress signal recognition and subsequent molecular, biochemical, and physiological responses activated through signal transduction (Komatsu et al., 2009; Ge et al., 2010; Le et al., 2012). Understanding such responses is very important for effective management of abiotic stress. Transcriptome profiling provides an opportunity to investigate plant response regulation and to identify genes involved in stress tolerance mechanisms. Earlier, approaches using expressed sequence tags (ESTs) sequencing along with several techniques, such as suppression subtractive hybridization (SSH), have been extensively used for transcriptome profiling of soybean under abiotic stress conditions (Clement et al., 2008). In addition, information of ESTs have been used to develop spotted microarrays (O'Rourke et al., 2007). These techniques are efficient but do not ensure analysis of entire genes in the soybean genome. Several high-throughput techniques have been developed for transcriptome analysis due to the advancement in sequencing technology and the availability of the whole soybean genome sequence, (Libault et al., 2010; Schmutz et al., 2010; Cheng et al., 2013). These platforms have been extensively used for transcriptome profiling to uplift abiotic stress tolerance mechanisms in soybean (Table 3).

**Table 3:** **Major transcriptomic analysis for the abiotic stress tolerance in soybean using different technological platforms**.

**Sr. No**.	**Trait/tissue**	**Platform**	DEG^*^	**Key points**	**References**
1	Soybean root development/root tips and non-meristematic tissue	Affymetrix chips containing 37,500 probe sets	9148	Resource of novel target genes for further studies involving root development and biology	Haerizadeh et al., 2011
2	Iron stress/root from isogenic lines	Custom array containing 9728 cDNAs	48	Genes involved in DNA repair and RNA stability were induced	O'Rourke et al., 2007
3	Drought stress at late developmental stages/V6 and R2 stages under drought and control	61 K Affymetrix Soybean Array GeneChip	3276 for V6 3270 for R2	Expression of many *GmNAC* and hormone-related genes was altered by drought in V6 and/or R2 leaves	Le et al., 2012
4	Herbicide resistance/plant under atrazine and bentazon stress	cDNA microarray with 36,760 different cDNA clones	6646	Expression of genes related to cell recovery, such ribosomal components	Zhu et al., 2009
5	Saline-alkaline stress tolerance/NaCl and NaHCO_3_ treatments	AffymetrixSoybean GeneChip	9027	Genes with altered expression regulated by alkaline stress	Ge et al., 2010
6	Flooding stress	HiCEP (29,388) high coverage expression profiling	97 genes and 34 proteins	Combined approach with proteomics	Komatsu et al., 2009

* Differentially expressed genes.

Microarray is a high-throughput technology where thousands of probes representing different genes are hybridized with RNA samples. Using the hybridization signal level, gene expression is calculated. The Affymetrix GeneChip representing 61K probe sets is routinely being used for transcriptome profiling of soybean under different abiotic stresses (Haerizadeh et al., 2011; Le et al., 2012). The normalized expression data generated using the Affymetrix GeneChip can be used to compare soybean experiments performed across the world. An expression database has been developed to globally explore public and proprietary expression data (www.genevestigator.com). The microarray data represents various tissues, developmental stages, and environmental conditions (Table 3). Effective analysis of such tremendous data using sequence homology and functional annotation will be helpful to understand biological processes.

### RNA-seq, an advanced approach for transcriptome profiling

Cost effective and high-throughput sequencing technologies make it possible to analyze transcriptomes by sequencing, known as RNA-seq. The RNA-seq approach has several advances over the microarray technology where available genomic information is used to design probe sets. However, RNA-seq does not require gene information and is capable of identifying novel transcripts that were previously unknown and also provides opportunities to analyze non-coding RNAs. The relative accuracy of microarrays and RNA-Seq has been evaluated using proteomics and it has been shown that RNA-Seq provides a better estimate of absolute expression levels (Fu et al., 2009). Applications of RNA-seq can be expanded further with an increased understanding of molecular regulations. For instance, RNA-seq is being used for transcription start site mapping, strand-specific measurements, gene fusion detection, small RNA characterization, and detection of alternative splicing events (Ozsolak and Milos, 2010).

RNA-Seq has been performed to investigate seven tissues and seven stages in seed development in soybean (Severin et al., 2010). This effort has generated an expression atlas for soybean genes which serves as a useful resource. The tissue specific expression pattern of genes is helpful in understanding regulation and tissue specific function.

### Combining QTL mapping, GWAS, and transcriptome profiling

QTL mapping and GWAS are very effective approaches to identify chromosomal region(s) associated with a particular phenotype. However, QTL spans large segments of chromosomes and it is also the same for GWAS where LD decay is slow as in case of soybean (Hyten et al., 2007). QTL or GWAS loci possess hundreds of genes that make the identification of candidate genes difficult (Sonah et al., 2012). This is similar in transcriptome profiling where thousands of genes have been found to be differentially expressed even with genetically similar isogenic lines (Table 3). Therefore combining QTL mapping or GWAS with transcriptome profiling will complement each other. For instance, candidate genes for grain number QTL in rice have been identified using microarray based transcriptome profiling of recombinant inbreed lines with contrasting phenotypes (Deshmukh et al., 2010; Sharma et al., 2011; Kadam et al., 2012). Similarly, a pair of soybean near-isogenic lines (NILs) differing in seed protein and an introgressed QTL segment (~8.4 Mb) have been used to study variation in transcript abundance in the developing seed (Bolon et al., 2010). The study identified 13 candidate genes in the QTL region using the Affymetrix Soy GeneChip and high-throughput Illumina whole transcriptome sequencing (Bolon et al., 2010). A combined approach of mapping and transcriptome profiling is based on an assumption that the quantitative trait is regulated by differential expression of candidate genes. This is not always true. Most of the time sequence variation present in candidate genes may cause defective proteins (Xu et al., 2013). Therefore, re-sequencing of QTL locus along with transcriptomics will also be a valuable approach to compliment mapping efforts.

## Proteomics in soybean

Proteomics deals with structural and functional features of all the proteins in an organism. It is important to understand complex biological mechanisms including the plant responses to abiotic stress tolerance. Abiotic stress tolerance mechanisms involve stress perception, followed by signal transduction, which changes expression of stress-induced genes and proteins. Post-translational changes are also important in plant responses to abiotic stresses. A single gene can translate in several different proteins and a few genes can lead to a diverse proteome. Such inconsistency limits genomics and transcriptomic approaches more specifically, when post translational changes govern phenotype. Differential expression observed at the transcriptional (mRNA) level need not be translated into differential amounts of protein. To address this, several proteomic studies have been performed to understand abiotic stress tolerance mechanisms in soybean (Table S4).

Unexpected levels of changes in the soybean proteome can occur during stress response and these changes can lead to different defense mechanisms. Some common proteins involved in redox systems, carbon metabolism, photosynthesis, signaling, and amino acid metabolism have been found to be associated with various stress responses in soybean (Zhen et al., 2007; Aghaei et al., 2009; Yamaguchi et al., 2010; Qin et al., 2013). These candidate proteins can directly link to genetic regulation of stress response in soybean. Candidate protein information can be used for the functional annotation of genes present in QTL regions or found differentially expressed under stress conditions.

In the near future, various proteomics approaches will be routinely used in soybean research that will generate tremendous information regarding structural and functional attributes of proteins. A systematic cataloging of information in the form of a publically accessible database is very important. Recently, a proteome database has been developed that contains reference maps of the soybean proteome collected from several organs, tissues, and organelles (Mooney and Thelen, 2004; Brechenmacher et al., 2009; Ohyanagi et al., 2012). Presently, these reference maps comprised information of about 3399 proteins from seven organs and 2019 proteins from four subcellular compartments that were identified using two-dimensional electrophoresis (http://proteome.dc.affrc.go.jp/soybean/). Volunteer deposition of proteomic information in such databases is necessary for effective utilization of available knowledge for the management of abiotic stress tolerance in soybean.

## Metabolomics advances for abiotic stress

Metabolomic studies in plants aim to identify and quantify the complete range of primary and secondary metabolites involved in biological processes. Therefore metabolomics provides a better understanding of biochemical pathways and molecular mechanisms. The knowledge of genes, transcripts and proteins involved cannot alone help to understand the biological process completely until knowledge of metabolites that are involved becomes available.

Several metabolomics studies have been performed to understand biochemical processes in soybean (Table S5). Development of new chromatographic and mass spectrometric platforms along with the enhancement of operational and analytical capabilities of existing platforms revolutionizes metabolomic investigations both in plant and animal sciences. The platforms such as gas chromatography mass spectrometry (GC-MS), fourier transform ion cyclotron resonance mass spectrometry (FT-ICR-MS), liquid chromatography mass spectrometry (LC-MS), capillary electrophoresis mass spectrometry (CE-MS), and nuclear magnetic resonance (NMR) are routinely used in plant sciences (Putri et al., 2013). Capability, limitations and specificity of these techniques has been recently reviewed in terms of effective utilization of these advanced resources (Putri et al., 2013). In-depth accurate analyses of metabolite information including the spectral data are the major challenge for the use of high-throughput techniques. Several statistical models and bioinformatics programs have been developed to analyze the metabolome in an interactive manner (Fernie et al., 2011; Putri et al., 2013).

## Ionomics in soybean

Ionomics is the study of elemental composition of an organism that mostly deals with high-throughput identification and quantification. Ionomics is important to understand element composition and their role in biochemical, physiological functionality and nutritional requirements of plants. Phosphorus (P) and potassium (K) are the two key elements used as macronutrients in fertilizer to ensure better crop yield. However plants require many other elements and those are not uniformly distributed among different soil types. Plants have evolved with a diverse element uptake ability at different locations because of diverse soil types (Fujita et al., 2013). This justifies the need of integrating ionomics with genomics to explore existing genetic differences. An ionomic study has been performed to analyze concentrations of 17 different elements in diverse accessions and three RIL populations of Arabidopsis thaliana grown in several different environments (Buescher et al., 2010). Significant differences in elemental composition between the Arabidopsis accessions were detected and more than hundred QTL were identified for different elemental accumulation (Buescher et al., 2010). Most of the ionomics studies to date in soybean have been performed to analyze nutritive value of soybean products (Table S6).

The elemental composition of a plant is controlled by multiple factors including element availability, uptake capability of roots, transport, and external environment which regulate physiological processes such as evapotranspiration. Because of such factors, the plant ionome has become very sensitive and specific so that the element profile reflects different physiological states. Recently a study performed in barley has analyzed ionome of wild accessions and cultivar differing in salt tolerance, grown in presence of 150 and 300 mM NaCl (Wu et al., 2013) and observed decreased amounts of K, magnesium (Mg), P and manganese (Mn) in roots and K, calcium (Ca), Mg and Sulfur (S) in shoots at the seedling stage. In addition, significant negative correlation among the amount of accumulated Na and metabolites involved in glycolysis and tricarboxylic acid (TCA) cycle have been observed (Wu et al., 2013). This ionomic study suggests the possible rearrangement of elemental profiles and metabolic processes to modify the physiological mechanisms of salinity tolerance.

Improvement in abiotic stress tolerance with the application of several inorganic element has been observed (Liang et al., 2007; Pilon-Smits et al., 2009). For instance, silicon (Si) has shown beneficial effects against different abiotic stresses including high salinity, water stress, heavy metal stress, and UV-b (Liang et al., 2007). Previously, soybean has been considered as poor accumulator of silicon mostly because of the genetic differences existing in the germplasm and very few genotypes have been evaluated to draw this conclusion (Hodson et al., 2005). However, with the advancement in ionomics technologies, silicon transporter genes have been identified recently in soybean using the integrated omics approach (Deshmukh et al., 2013). This study has used computational genomics, transcriptomics, and ionomics information available in the model plant species such as Arabidopsis and rice. Besides this, high-throughput efforts for maximum number of elemental profiles in soybean in respective external environment are required. That will definitely improve the understanding of the soybean ionome and its subsequent utilization in the management of abiotic stress tolerance.

## Phenomics prospective in soybean

**Figure 3:**
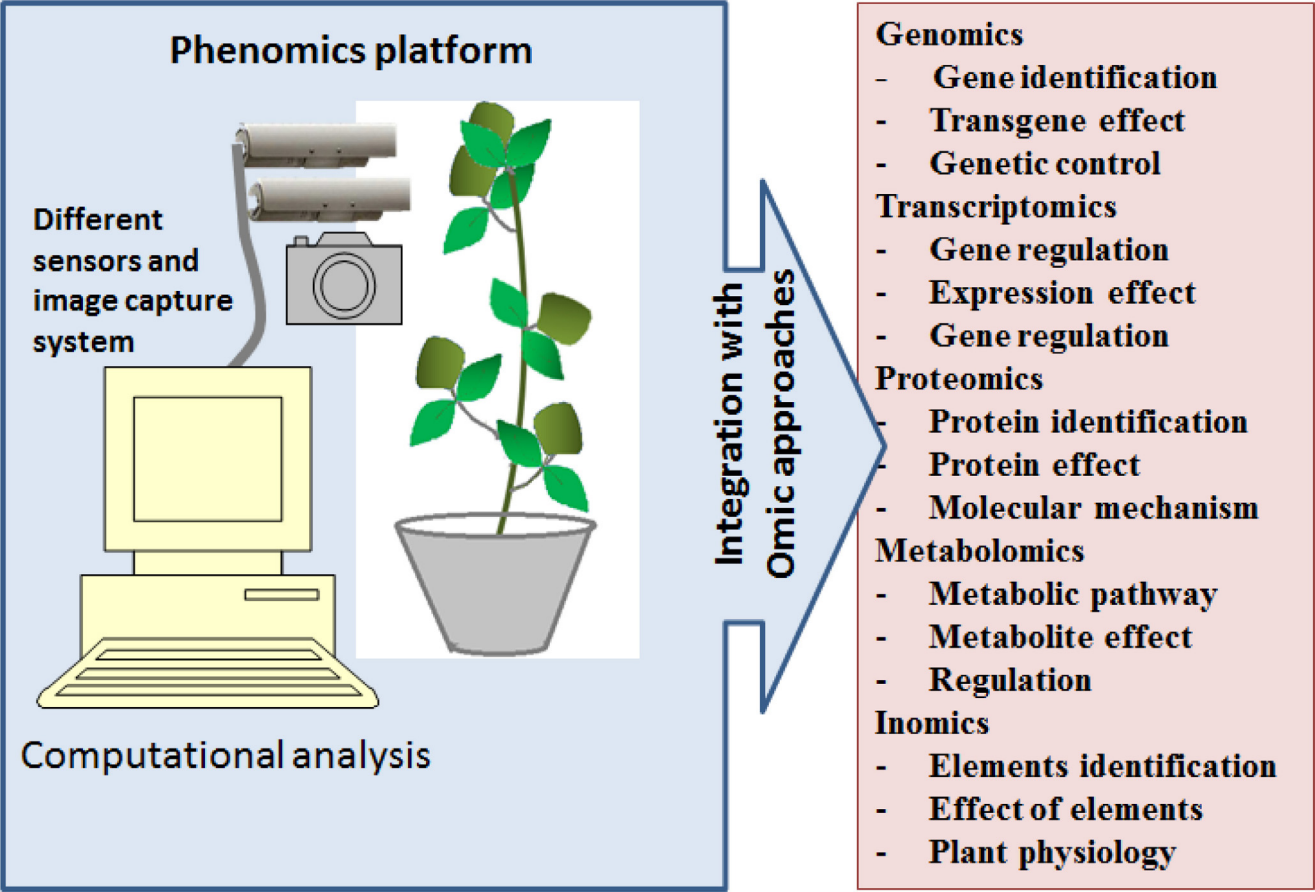
**Phenomics and its integration with other omics approaches**.

The phenotype is a physical and biochemical trait of an organism. Phenomics is a study involving high-throughput analysis of phenotype. Phenotype is the ultimate resultant from the complex interactions of genetic potential between an organism and environment. Precision phenotyping is important to understand any biological system. In plant as well as animal sciences, a particular phenotype (as symptoms) is used to understand biological status, such as disease, pest infestation or physiological disorders. With technological advances, genomic resources have been routinely used to predict phenotype based on the evaluation of genetic markers; it can be called “genetic symptoms.” The success of genomics is based on how reliable connection is there between a genetic marker and the phenotype. In plant breeding, genetic improvement through omics approaches is being conducted to achieve ideal phenotype that will ensure higher and stable yield under diverse environmental conditions. Therefore phenomics integrated with other omics approaches has the most potential in the plant breeding (Figure 3).

Phenome has a broader meaning than what is being generally considered. It is not limited to the visible morphology of an organism but expectedly larger and complex. Unlike genomics, where the entire genome can be characterized by sequencing, the phenome cannot be characterized entirely. Therefore, the term phenomics being an analogy to genomics expected only study of particular set of phenotype at high-throughput level and not the entire set. In this regards, the technological development in image processing and the automation techniques have played important roles. Plant imaging with light sources from visible to near infrared spectrum provides an opportunity for non-destructive phenotyping. Therefore, real-time analysis of plant development became possible. Moreover, robotic technologies used in phenomic platforms have increased the precision and speed of phenotyping. This has allowed for incorporating additional aids such as precise irrigation and fertilization systems. For instance, “PHENOPSIS” an automated phenomic platform has been developed to study water stress in Arabidopsis and has a robotic arm loaded with a tube for irrigation and a camera (Granier et al., 2006). These types of advanced phenomic platforms have been developed and made available for wider range of crop plants (www.lemnatec.com). However, these platforms have not gained the expected popularity even though tremendous advancement in both imaging as well as robotic technology has been achieved.

In soybean, several phenomic efforts have been performed but most of these are pilot experiments (Table S7). Recently, a method has been developed to assess leaf growth in soybean under different environmental conditions (Mielewczik et al., 2013). This method can utilize different light sources that are available in a greenhouse as well as under field conditions. Marker tracking approaches (Martrack Leaf) have also been used to facilitate accurate analysis of two-dimensional leaf expansion with high temporal resolution (Mielewczik et al., 2013). Apart from this, phenomics has been used to facilitate efficient identification of soybean cultivars which is very important for germplasm resource management and utilization (Zhu et al., 2012). Zhu et al. (2012), used a laser light back-scattering imaging technology to analyze single seed. Images of laser light illuminated the soybean seed surface were captured by a charge-coupled device (CCD) camera. The characteristic pattern of laser luminance is analyzed by image processing technology to identify a particular cultivar. Such characteristic of laser light back-scattering can be used to assess quality and other seed characteristics as markers for selection in breeding programs.

Phenomics in soybean is lagging far behind genomics because hundreds of genomes and many genetic populations are re-sequenced. One best example is the 1000 genome re-sequencing project at the University of Missouri, MO, USA (http://soybeangenomics.missouri.edu/news2012.php). The 1000 genome project will generate a huge amount of genomic information which will require utilization of comparable phenomic data. This will be helpful to accelerate soybean research in many ways.

## Role of online databases for effective integration of omics platforms

The recent advancement in the omic platforms has generated tremendous information which has been used to promote research activities in all possible dimensions. Utilization of available information has become possible because of computational resources that helps to catalog, store, and analyze available data and make it easily accessible through user friendly interfaces so called “databases.” In this regard, several databases have been developed for soybean (Table 4). Among these, Soybean Knowledge Base (SKB, http://soykb.org) is a very useful database that provides a comprehensive web resource for omics data from several different platforms (Joshi et al., 2012). The SKB resources are helpful for bridging soybean translational genomics and molecular breeding research. It contains information of genes, proteins, microRNAs, sRNAs, metabolites, molecular markers, and phenomic information of soybean plant introductions (PI). It also provides interference to integrate multi-omics datasets and because of this, a galaxy of information becomes comparable and more useful. For instance, genes in the QTL region can be retrieved very easily along with the functional annotations, associated protein information in respect of structure and functional features, syntenic information with other model plants, sequence variation among different cultivars, gene expression data including tissue specific variations and many other types of information for soybean.

**Table 4:** **Online databases exclusively developed to host soybean research data generated from different omics platforms**.

**Sr. No**	**Database**	**Features**	**Tools**
1	SoyBase	Genetic and physical maps, QTL, Genome sequence, Transposable elements, Annotations, Graphical chromosome visualizer	BLAST search, ESTs search, SoyChip Annotation Search, Potential Haplotype (pHap) and Contig Search, Soybean Metabolic Pathways, Fast Neutron Mutants Search, RNA-Seq Atlas
	SoyBase and the Soybean Breeder's Toolbox, USDA and Iowa University, http://soybase.org/		
2	SoyKB	Multi-omics datasets, Genes/proteins, miRNAs/sRNAs, Metabolite profiling, Molecular markers, information about plant introduction lines and traits, Graphical chromosome visualizer	Germplasm browser, QTL and Trait browser, Fast neutron mutant data, Differential expression analysis, Phosphorylation data, Phylogeny, Protein BioViewer, Heatmap and hierarchical clustering, PI and trait search, FTP/data download capabilities
	Soybean Knowledge Base, University of Missouri, Columbia, http://soykb.org/		
3	SoyDB	Protein sequences, Predicted tertiary structures, Putative DNA binding sites, Protein Data Bank (PDB), Protein family classifications	PSI-BLAST, Browse database, Family Prediction by HMM, FTP data retriever
	Soybean transcription factors database, Missouri University, http://casp.rnet.missouri.edu/soydb/		
4	SGMD	Integrated view genomic, EST and microarray data	Analytical tools allowing correlation of soybean ESTs with their gene expression profiles
	The Soybean Genomics and Microarray Database, http://bioinformatics.towson.edu/SGMD/		
5	Deltasoy	Official variety trial (OVT) information in soybean, Mississippi OVT data, including yield, location, and disease information	Comparison tools for variety trail data, phenotypic data and disease related data
	An Internet-Based Soybean Database for Official Variety Trials, http://msucares.com/deltasoy/testlocationmap.htm		
6	DaizuBase	BAC-based physical map, Linkage map and DNA markers, BAC-end, BAC contigs, ESTs, full-length cDNAs	Gbrowse, Unified Map, Gene viewer, BLAST
	An integrated soybean genome database including BAC-based physical maps, http://daizu.dna.affrc.go.jp/		
7	SoyMetDB	Soybean metabolomic data	Pathway Viewer
	The soybean metabolome database, http://soymetdb.org		
9	SoyProDB	Several 2D Gel images showing isolated soybean seed proteins	Search tool for 2D spots, Navigation tools for protein data
	Soybean proteins database, http://bioinformatics.towson.edu		
10	SoyGD	Physical map and genetic map, Bacterial artificial chromosome (BAC) fingerprint database, Associated genomic data	Sequence data retrieval tools, Navigation tool for sequence information of different builds
	The Soybean GBrowse Database, Southern Illinois University, http://soybeangenome.siu.edu/		
11	SoyTEdb	Williams 82 transposable element database	Browse for Repetitive elements, Transposable Element and Map position, Data retrieval tools
	Soybean transposable elements database, www.soybase.org/soytedb/		
12	SoyXpress	Soybean ESTs, Metabolic pathways, Gene Ontology terms, Swiss-prot Identifiers and Affymetrix gene expression data	BLAST search, Microarray experiments, Pathway search etc
	Soybean transcriptome database, http://soyxpress2.agrenv.mcgill.ca		

## General conclusion

Different omics tools have been employed to understand how soybean plants respond to abiotic stress conditions. We realize that the studies to integrate multiple omics approaches are limiting in soybean due to the increased cost and potential challenging integrated omic scale analysis. Recent developments in computational resources, statistical tools, and instrumentation have lowered the cost of omics in many folds but integrated analysis needs novel tools and technical wizards. The comprehensive nature of multi-omic studies provides an entirely new avenue and future research programs should plan to adapt accordingly. In soybean, genomics and transcriptomics have progressed as expected but the other major omic branches like proteomics, metabolomics, and phenomics are still lagging behind. These omic branches are equally important to get clear picture of the biological system. Notably, phenomic studies need to be extensively employed along with the other omics approaches. Desired phenotype is ultimate aim of crop sciences; therefore it needs to be understood intensely. Different omic tools and integrated approaches discussed in the present review will provide glimpses of current scenarios and future perspectives for the effective management of abiotic stress tolerance in soybean.

### Conflict of interest statement

The authors declare that the research was conducted in the absence of any commercial or financial relationships that could be construed as a potential conflict of interest.

## Footnotes

^1^Available online at: http://www.soystats.com (Accessed December 10, 2013).
