# Supplementary material for: Integrating omic approaches for abiotic stress tolerance in soybean
Source: Front Plant Sci. 2014 Jun 3;5:244. doi: 10.3389/fpls.2014.00244 (PMC4042060; doi:10.3389/fpls.2014.00244)
Supplement: Supplementary file 1 [file DataSheet1.DOCX]

**Supplementary Material**

**Table S1** Details of QTL mapping studies performed for the elevation of different abiotic stress tolerance in soybean

| **Traits** | **Parents** | **Population size** | **Population type** | **Methods** | **QTLs** | **Reference** |
| --- | --- | --- | --- | --- | --- | --- |
| **Lodging** | G. max 7499 x G. soja PI 245331 | 120 | RIL | CIM | 1 | ([Li et al., 2008](#_ENREF_54)) |
|  | PIs x Beeson/ kenwood/Lawrence | 236 | RIL | CIM | 7 | ([Guzman et al., 2007](#_ENREF_25)) |
|  | Zhongdou29 x Zhongdou32 | 165 | RIL | CIM | 6 | ([Rong et al., 2009](#_ENREF_79)) |
|  | Essex x Forrest | 100 | RIL | CIM | 1 | ([Kassem et al., 2006](#_ENREF_43)) |
|  | A5403 x Archer, | 103 | RIL | SMA, CIM | 17, 1 | ([Cornelious et al., 2005](#_ENREF_14)) |
|  | P9641 x Archer | 67 | RIL | SMA, CIM | 15, 1 | ([Cornelious et al., 2005](#_ENREF_14)) |
| **Drought** | Kefeng1 x Nannong1138-2 | 184 | RIL | CIM | 10 | ([Du et al., 2009a](#_ENREF_18)) |
|  | Hongfeng 11 x Harosoy | 95 | BC2F3 | ANOVA | 18 | ([Zhang et al., 2012](#_ENREF_98)) |
|  | Kefeng1 x Nannong1138-2 | 184 | RIL | CIM | 17 | ([Du et al., 2009b](#_ENREF_19)) |
|  | AC Colibri×OT91-3 | 200 | RIL | SIM, sCIM | 5 | ([Molnar et al., 2012](#_ENREF_66)) |
|  | PI 416937 x Benning | 147 | RIL | MIM | 4 | ([Carpentieri-Pipolo et al., 2012](#_ENREF_8)) |
|  | S-100 × Tokyo, | 116 | F 2 | IM | 2 | ([Mian et al., 1998](#_ENREF_64)) |
|  | Young × PI416937, | 120 | F 4 | ANOVA | 5 | ([Mian et al., 1996](#_ENREF_65)) |
|  | Jackson × KS4895 | 81 | RILs | IM | 1 | ([Bhatnagar et al., 2005](#_ENREF_6)) |
| **Salt** | Kefeng1 x Nannong1138-2 | 184 | RIL | CIM | 8 | ([Chen et al., 2008](#_ENREF_9)) |
|  | PI 483463 x Hutcheson |  | RIL |  | 1 | ([Ha et al., 2013](#_ENREF_26)) |
|  | Jackson x JWS156-1 | 1,109 | RIL |  | validation | ([Tuyen et al., 2013](#_ENREF_85)) |
|  | Jackson x JWS156-1 | 225 | F2 | CIM | 2 | ([Hamwieh and Xu, 2008](#_ENREF_29)) |
|  | S-100 x Tokyo | 106 | F2:5 | ANOVA | 1 | ([Lee et al., 2004](#_ENREF_53)) |
|  | FT-Abyara x C01 | 96 | RIL | CIM | 1 | ([Hamwieh et al., 2011](#_ENREF_28)) |
|  | Jin dou No. 6 × 0197 | 81 | RIL | CIM | 1 | ([Hamwieh et al., 2011](#_ENREF_28)) |
| **Manganese toxicity** | Essex x Forrest | 100 | RILs | ANOVA, CIM | 3 | ([Kassem et al., 2004](#_ENREF_44)) |
| **Phosphorus deﬁciency** | Kefeng 1 x Nanong 1138-2 | 184 | RILs | CIM | 7 | ([Li et al., 2005](#_ENREF_55)) |
| **Phosphorus** | Anoka x A7 | 92 | F2:4 | IM, MQM | 1 | ([King et al., 2013](#_ENREF_46)) |
| **Temperature** | Hongfeng 11 x Harosoy | 95 | BC2F3 | ANOVA | 23 | ([Zhang et al., 2012](#_ENREF_98)) |
| **Iron** | Anoka x A7 | 92 | F2:4 | IM, MQM | 1 | ([King et al., 2013](#_ENREF_46)) |
| **Aluminum** | Kefeng1 xNannong1138-2 | 184 | RIL | MCIM | 9 | ([Korir et al., 2011](#_ENREF_51)) |
| **Aluminum** | Young x PI229358 |  | F4 | ANOVA | 6 | ([Bianchi-Hall et al., 2000](#_ENREF_7)) |
| **Aluminum** | Essex x Forrest | 42 | RIL | ANOVA, CIM | 3 | ([Sharma et al., 2011](#_ENREF_81)) |

**Table S2** Details of genome-wide association studies (GWAS) performed for different traits in soybean

| **Sr. No.** | **Trait** | **GWAS loci** | **Markers** | **Genotypes** | **Method** | **References** |
| --- | --- | --- | --- | --- | --- | --- |
| 1 | Iron deficiency | 3 | SSR |  | SFA, MLM, | ([Wang et al., 2008](#_ENREF_89)) |
| 2 | Iron deficiency | 42  88 | 858 SNP 868 SNP | 143  141 | MLM, GLM | ([Mamidi et al., 2011](#_ENREF_59)) |
| 3 | Soybean cyst nematode  Soybean mosaic virus  Salt tolerance  Cold tolerance  Drought tolerance  Seed oil content  Seed protein content | 6  3  -  -  5  6  1 | 55 SSR | 159 |  | ([Li et al., 2011](#_ENREF_56)) |
| 4 | Seed size and shape traits | 59 | 135 SSR | 257 | E-cMLM | ([Niu et al., 2013](#_ENREF_69)) |
| 5 | Aluminum tolerance using relative root elongation | 11 | 197 SSR | 188 | MLM | ([Korir et al., 2013](#_ENREF_52)) |
| 6 | Response to photoperiod and temperature | 9 | 118 SSR | 275 | GLM | ([Zuo et al., 2013](#_ENREF_103)) |
| 7 | Yield and yield components | 19 | 1536 SNP | 191 | MLM | ([Hao et al., 2012b](#_ENREF_32)) |
| 8 | Chlorophyll and chlorophyll fluorescence parameters | 51 | 1536 SNP | 168 | MLM | ([Hao et al., 2012a](#_ENREF_31)) |
| 9 | seed protein  seed oil | 17  13 | 55159 SNP | 298 | MLM | ([Hwang et al., 2014](#_ENREF_37)) |
|  |  |  |  |  |  |  |

Single factor analysis of variance (SFA), mixed linear model (MLM), enriched compression mixed linear model (E-cMLM), generalized linear model (GLM)

**Table S3** Details of significant efforts of genomic selection performed using different prediction models in different crop species.

| **Species** | **Population type** | **Population size** | **Total markers** | **Accuracy of GEBVs** | **Models used for GEBV** | **Traits** | **Reference** |
| --- | --- | --- | --- | --- | --- | --- | --- |
| Wheat | CIMMYT lines | 599 | 1279 DArTs | 0.48-0.61 | PM-RKHS | Grain yield | ([Crossa et al., 2010](#_ENREF_15)) |
| Maize | CIMMYT lines | 300 | 1148 SNPs | 0.42–0.79 | M-BL | Grain yield, female ﬂowering, male ﬂowering, anthesis-silking interval | ([Crossa et al., 2011](#_ENREF_16)) |
| Eucalyptus | 75 full-sib family 55 elite parents | 920 | 3564 DArTs | 0.54–0.62 | BLUP | Height, diameter at breast height, wood density, pulp yield, lignin content, Puccinia rust resistance | ([Grattapaglia and Resende, 2011](#_ENREF_23)) |
| Eucalyptus | 43 full-sib family 11 interspeciﬁc hybrid | 783 | 3120 DArTs | 0.53–0.69 | BLUP | Height, diameter at breast height, wood density, pulp yield, lignin content, Puccinia rust resistance | ([Grattapaglia et al., 2012](#_ENREF_24)) |
| Arabidopsis thaliana | RILs | 415 | 69 SSR | 0.90–0.93 | BLUP | Flowering time, dry matter, free amino acid | ([Lorenzana and Bernardo, 2009](#_ENREF_57)) |
| Barley | DHLs | 150 | 223RFLP | 0.64-0.83 | BLUP | Plant height, grain yield, 3 chemical components | ([Lorenzana and Bernardo, 2009](#_ENREF_57)) |
| Barley | DHLs | 140 | 107 RFLP and AFLP | 0.66-0.85 | BLUP | Plant height, two chemical components | ([Lorenzana and Bernardo, 2009](#_ENREF_57)) |
| Maize | F2 | 349 | 160 SSR | 0.59-0.72 | BLUP | 3 morphological traits, grain moisture | ([Lorenzana and Bernardo, 2009](#_ENREF_57)) |
| Maize | Testcrosses of DHLs | 371 | 125SNPs | 0.31-0.55 | BLUP | 3 morphological traits, grain moisture | ([Lorenzana and Bernardo, 2009](#_ENREF_57)) |
| Maize | RILs | 223 | 1339SSR and RFLP | 0.48-0.73 | BLUP | 8 morphological traits, 3 chemical components, grain moisture | ([Lorenzana and Bernardo, 2009](#_ENREF_57)) |
| Maize | RILs | 119 | 1339SSR and RFLP | 0.40-0.50 | BLUP | 5 morphological traits, grain moisture | ([Lorenzana and Bernardo, 2009](#_ENREF_57)) |
| Wheat | DHLs | 209 | 399 SSRs, DArTs, AFLPs, TRAPs, STS | 0.32–0.84 | RR-BLUP | 8 grain quality | ([Heffner et al., 2011](#_ENREF_33)) |
| Wheat | DHLs | 174 | 574 DArTs | 0.41–0.73 | RR-BLUP | 8 grain quality | ([Heffner et al., 2011](#_ENREF_33)) |
| Loblolly pine | 61 full-sib families | 790 – 840 | 3938 SNPs | 0.64–0.77 | BLUP | Diameter at breast height, total height | ([Resende et al., 2011](#_ENREF_74)) |
| Loblolly pine | Full-sib offspring | 149 | 3406 SNPs | 0.3–0.83 | Pedigree model | Growth and quality traits | ([Isik et al., 2011](#_ENREF_38)) |
| Loblolly pine | 70 full-sib families | 951 | 4853 SNPs | 0.37-0.77 | RR–BLUP, Bayes A, Bayes Cπ, Bayesian LASSO | 17 traits | ([Resende et al., 2012b](#_ENREF_76)) |
| Loblolly pine | 50 haploids | 769 | 4755 SNP | 0.33-0.94 | GBLUP, BayesA, and BayesCp | disease resistance Rust and Rust gall volume | ([Jia and Jannink, 2012](#_ENREF_42)) |
| Loblolly pine | 61 full-sib families | 800 | 4825 SNP | 0.63-0.75 | BLUP | diameter and height | ([Resende et al., 2012a](#_ENREF_75)) |
| Wheat | Commercial lines | 139 | 2395 SNPs | 0.175-0.514 | G-BLUP | grain yield | ([Jarquín et al., 2013](#_ENREF_41)) |
| Wheat | Elite lines | 306 | 1717 DArT | 0.20-0.75 | Bayesian LASSO, Bayesian ridge regression, | Days to heading and grain yield | ([Pérez-Rodríguez et al., 2012](#_ENREF_72)) |
| Maize | Inbred lines | 289 | 56,110 SNPs | 0.45-0.82 | RR-BLUP, LASSO | 3 agronomic traits and 3 metabolites | ([Riedelsheimer et al., 2012](#_ENREF_78)) |
| Maize | DHLs | 177 | 768 SNP | 0.476 to 0.710 | RR-BLUP | grain yield | ([Schulz-Streeck et al., 2013](#_ENREF_80)) |
| Sugarcane | Accessions (Reunion Island) | 167 | 1499 DArT | 0.29 - 0.62 | LASSO | 10 Traits | ([Gouy et al., 2013](#_ENREF_22)) |
| Sugarcane | Accessions (Guadeloupe) | 167 | 1499 DArT | 0.11 - 0.5 | LASSO | 10 Traits | ([Gouy et al., 2013](#_ENREF_22)) |
| Sugar beet | Elite inbred lines | 924 | 677 SNP | 0.48-0.80 | RR-BLUP | 6 Traits | (Würschum et al., 2013) |
| Sugar beet | inbred lines derived from 34 crosses | 310 | 384 SNP | 0.4-0.86 | RR-BLUP | sugar content and loss to molasses | ([Hofheinz et al., 2012](#_ENREF_34)) |
| Rapeseed | DHLs | 391 | 253 SNP | 0.41-0.84 | RR-BLUP | 6 Traits | ([Würschum et al., 2014](#_ENREF_92)) |
| Pear | cultivars | 76 | 155SSRs, 4 RAPD-STS | 0.2-0.75 | Bayesian regression | 9 Traits | ([Iwata et al., 2013](#_ENREF_40)) |
| Soybean | Accessions (Guadeloupe) | 288 | 79 SCAR | 0.69-0.904 | RR-BLUP, BLR | HSW | ([Shu et al., 2012](#_ENREF_82)) |
| Soybean | RILs | 126 | 80SSRs | 0.12- 0.78 | Empirical Bayesian method | Primary embryogenesis capacity | ([Hu et al., 2011](#_ENREF_36)) |

**Table S4** Details of Proteomic studies performed for different traits in soybean

| **Sr. No.** | **Traits/ Tissue samples** | **Technique** | **Spots/ Candidate Proteins** | **References** |
| --- | --- | --- | --- | --- |
| 1 | Seed Filling/Developing seeds | 2D-PAGE,MALDI-TOF | 679 spots/422Proteins/216NR Proteins | ([Hajduch et al., 2005](#_ENREF_27)) |
| 2 | Seed filling | 2-DGE, Sec-MudPIT, LCMS | 531 spots/ 478 NR Proteins | ([Agrawal et al., 2008](#_ENREF_2)) |
| 3 | Soybean peribacteroid membrane (PBM) proteins | 2D-PAGE | 200 spots/17 proteins | ([Panter et al., 2000](#_ENREF_71)) |
| 4 | Two major storage proteins, β-conglycinin and glycinin/ wild and cultivated soybean seeds | 2D-PAGE | 44 and 34 Proteins | ([Natarajan et al., 2006](#_ENREF_68)) |
| 5 | Aluminum-stress-responsive proteins | 2D-PAGE | 1200 spots/39 proteins | ([Zhen et al., 2007](#_ENREF_100)) |
| 6 | Salt stress/ hypocotyls and roots | 2D-PAGE | 321 spots/ 7 proteins | ([Aghaei et al., 2009](#_ENREF_1)) |
| 7 | Soybean root hairs after inoculation with *B. japonicum* | 2D-PAGE,MALDI-TOF, tandem MS (MS/MS) | 96 spots , 37 spots/ 27 Proteins | ([Wan et al., 2005](#_ENREF_88)) |
| 8 | Flavonoids against UV-B | 2-D PAGE, MS | 300 spots /67Proteins | ([Xu et al., 2008](#_ENREF_94)) |
| 10 | Salinity/ leaves, hypocotyls and roots | 2-D PAGE, MS | 340, 330 and 235 spots/ 19, 22 and 14 proteins | ([Sobhanian et al., 2010](#_ENREF_83)) |
| 11 | Seed proteins | 2DE, MALDI-TOF MS | 150spots/44Proteins | ([Mooney and Thelen, 2004](#_ENREF_67)) |
| 12 | Waterlogging stress/roots | 2-DE, MALDI-TOF, ESI-MS/MS | 900 spots/ 24 Proteins | ([Alam et al., 2010](#_ENREF_3)) |
| 13 | Germinating soybean seeds | LC-MS/MS | 764 Proteins | ([Han et al., 2013](#_ENREF_30)) |
| 14 | Flooding stress/Root and cotyledon | 2D-PAGE/nano LC, (MS)/MS | 615 and 377 spots/ 73 and 28 Proteins | ([Komatsu et al., 2013](#_ENREF_48)) |
| 15 | Cold and osmotic stress | LC/nanoESI-MS | 1272 spots/ 59 Proteins | ([Swigonska and Weidner, 2013](#_ENREF_84)) |
| 16 | Abiotic stress | iTRAQ /LC-MS/MS | 1269 NR Proteins | ([Qin et al., 2013](#_ENREF_73)) |
| 17 | Biotic and abiotic treatments | 2-DE, MALDI-TOF/TOF | 21 Proteins | ([Zhao et al., 2013](#_ENREF_99)) |
| 18 | Salt stress | 2D-PAGE, MALDI-TOF-MS | 350 pH range 3 to 10 and 650 spots pH range 4 to 7 / 18 Proteins | ([Xu et al., 2011](#_ENREF_95)) |
| 19 | Cadmium stress | 2-DE, MS | 15 spots /12 proteins | ([Hossain et al., 2012](#_ENREF_35)) |
| 20 | Ozone-induced responses | 2DE/ nanoLC-MS/MS | 1455 spots/277 Proteins | ([Galant et al., 2012](#_ENREF_21)) |
| 21 | Water stress | MALDI-TOF MS/MS | 1000 spots/35 Proteins | ([Yamaguchi et al., 2010](#_ENREF_96)) |

**Table S5** Details of significant efforts of metabolomics performed using different technological platforms in soybean.

| **Sr.**  **No** | **Trait** | **Methods** | **Metabolites** | **Reference** |
| --- | --- | --- | --- | --- |
| 1 | Mature leaves | NAQF) and GC-MS | 100 | ([Benkeblia et al., 2007](#_ENREF_5)) |
| 2 | Flooding | CE-MS | 81 | ([Komatsu et al., 2009](#_ENREF_49)) |
| 3 | Salt tolerance | HPLC-UV-ESI-MS | 200 | ([Wu et al., 2008](#_ENREF_91)) |
| 4 | Defense-related prenylated isoflavones | LC-MS and NMR | 26 | ([Cheng et al., 2011](#_ENREF_10)) |
| 5 | Defense response | LC-MS and GC-MS | - | ([Choi et al., 2010](#_ENREF_11)) |
| 6 | Soybean seed | UHLC/MS/MS2 | 169 | ([Clarke et al., 2013](#_ENREF_12)) |
| 7 | Seed filling | GC-MS/ UPLC/ FLD | 55 | ([Collakova et al., 2013](#_ENREF_13)) |
| 8 | Salt stress | GC–MS/LC–FT/MS | 98 | ([Lu et al., 2013](#_ENREF_58)) |
| 9 | Gamma-irradiated and non-irradiated soybeans | ^1^H NMR spectra | - | ([Ribeiro et al., 2014](#_ENREF_77)) |
| 10 | Fermentative Capability of Soybean Starter | ^1^H NMR spectra | - | ([Ko et al., 2010](#_ENREF_47)) |

**Table S6** Details of significant efforts of ionomics performed using different technological platforms in soybean.

| **Sr. No** | **Plant material** | **Platform/technology** | **Important note** | **Reference** |
| --- | --- | --- | --- | --- |
| 1 | soybean flour extract | ICP – AES  and ICP – MS | Iron is bound on high-molecular substances and its distribution is different from the phosphorus distribution. Manganese, copper and selenium occur in a wide range of molecular weights. | ([Fingerová and Koplík, 1999](#_ENREF_20)) |
| 2 | soybean flour and common white bean seeds extract | SEC and ICP-MS | Similar elution profiles of P, Fe, Co, Ni, Cu, Zn and Mo and significantly different Mn, Fe, Co and Se, the element profiles of soybean flour and white bean seed extracts | ([Koplı́k et al., 2002](#_ENREF_50)) |
| 3 | Soybean mutant screening | IC ICP-MS | Very high throughput effort screened seed elemental composition from a population of 947  NMU mutagenized soybean families | ([Ziegler et al., 2013](#_ENREF_102)) |
| 4 | Transgenic and non-transgenic soybean seeds | Size-exclusion [ICP-MS](javascript:popupOBO('CMO:0000538','c0mt00040j')) | Statistically significant differences in concentrations of Cu, Fe and Sr, which are also reflected by element contents in water extracts and residues | ([Mataveli et al., 2010](#_ENREF_61)) |
| 5 | common white bean, pea, chick pea seeds and defatted soybean flour | SEC/ICP-MS | Quantification of elements in the individual chromatographic fractions was carried out by isotope dilution and external calibration techniques. | ([Mestek et al., 2002](#_ENREF_63)) |
| 6 | Transgenic and non-transgenic soybean seeds | ICP-MS/ICP-AES | wholesome elements and heavy metals in ten soybean species from northeastern China were studied | ([Yan et al., 2007](#_ENREF_97)) |
| 7 | transgenic soybean oil | ICP-MS | Estimated concentration of 22 elements in transgenic soybean oil | ([Wel et al., 2008](#_ENREF_90)) |
| 8 | Soybean seed  and its products | ICP-MS | A method for bromine, chlorine, and iodine deterination in soybean and related products was developed by ICP-MS after digestion by microwave-induced combustion | ([Barbosa et al., 2012](#_ENREF_4)) |
| 9 | Transgenic and non-transgenic soybeans | 2D-HPLC-ICP-MS and ESI-MS/MS | Identified several metalloproteins in transgenic and non-transgenic soybeans | ([Mataveli et al., 2012](#_ENREF_60)) |
| 10 | Soybean from different climatic zones | ICP-AES | Results suggested that the contents of Al, CaFe, Na, Mn and Zn were easily affected by growth conditions, such as water, soil and so on. | ([WAN et al., 2010](#_ENREF_87)) |
|  |  |  |  |  |

Size exclusion chromatography (SEC), inductively coupled plasma mass spectrometry (ICP-MS), Inductively coupled plasma atomic emission spectroscopy (ICP-AES), Ion chromatography (IC)

**Table S7** Details of significant efforts of phenomics performed using different technological platforms in soybean.

| **Sr. No** | **Phenotypic parameters** | **Platform/technology** | **Important note** | **Reference** |
| --- | --- | --- | --- | --- |
| 1 | Soybean nodulation vs. Non-nodulation characteristic | Minolta SPAD spectrometer, digital still camera | Phenotyping of the soybean nodulation vs. non-nodulation characteristic with respect to leaf, agronomic and seed traits, and relating both chlorophyll and image analysis data to seed quality characteristics. | ([Vollmann et al., 2011](#_ENREF_86)) |
| 2 | Nitrate concentration in leaf | Near-infrared hyperspectral imaging system | A hyperspectral imaging system was developed to analyse nitrate metabolism. The model demonstrated a correlation coefficient of 0.9216 between the measured and estimated nitrate concentration | ([Matsuura et al., 2010](#_ENREF_62)) |
| 3 | Translocation of photosynthates to nodules | Positron-emitting tracer imaging system (PETIS) | Analysis of photosynthate translocation into the individual nodules in an intact soybean with real-time observation | ([Ito et al., 2010](#_ENREF_39)) |
| 4 | Soybean seed quality | Laser light backscattering imaging | The present study indicated that laser light scattering imaging was a promising method for the identification of the cultivars of single soybean seeds | ([Zhu et al., 2012](#_ENREF_101)) |
| 5 | Soybean rust | Multispectral CCD camera | two approaches to multispectral image processing for detecting soybean rust and its severity, either with or without manual threshold-setting, were investigated | ([Cui et al., 2010](#_ENREF_17)) |
| 6 | Water stress | Digital infrared thermography and thermometry | Canopy temperature data from infrared thermography were used to benchmark the relationship between an empirical crop water stress index (CWSIe) and leaf water potential (ΨL) across a block | ([O'shaughnessy et al., 2011](#_ENREF_70)) |

**A)**

**B)**

**Figure S1** Trend of research publications regarding genomic selection observed during last decade. A) number of scientific publications obtained by pubmed (http://www.ncbi.nlm.nih.gov/pubmed) search using “genomic selection” key word found only in abstract or title, and B) by google scholar (<http://scholar.google.ca/>) using “genomic selection” OR “genomic prediction” key word only in title. Search was performed on 23 December 2013

**References**

Aghaei, K., Ehsanpour, A., Shah, A., and Komatsu, S. (2009). Proteome analysis of soybean hypocotyl and root under salt stress. *Amino Acids* 36**,** 91-98.

Agrawal, G.K., Hajduch, M., Graham, K., and Thelen, J.J. (2008). In-depth investigation of the soybean seed-filling proteome and comparison with a parallel study of rapeseed. *Plant Physiol.* 148**,** 504-518.

Alam, I., Lee, D.-G., Kim, K.-H., Park, C.-H., Sharmin, S.A., Lee, H., Oh, K.-W., Yun, B.-W., and Lee, B.-H. (2010). Proteome analysis of soybean roots under waterlogging stress at an early vegetative stage. *J. Biosci.* 35**,** 49-62.

Barbosa, J.T.P., Santos, C.M.M., Dos Santos Bispo, L., Lyra, F.H., David, J.M., Korn, M.D.G.A., and Flores, E.M.M. (2012). Bromine, chlorine, and iodine determination in soybean and its products by ICP-MS after digestion using microwave-induced combustion. *Food Analytical Methods***,** 1-6.

Benkeblia, N., Shinano, T., and Osaki, M. (2007). Metabolite profiling and assessment of metabolome compartmentation of soybean leaves using non-aqueous fractionation and GC-MS analysis. *Metabolomics* 3**,** 297-305.

Bhatnagar, S., King, C.A., Purcell, L., and Ray, J.D. (2005). "Identification and mapping of quantitative trait loci associated with crop responses to water-deficit stress in soybean [Glycine max (L.) Merr.]", in: *The ASACSSA-SSSA International annual meeting poster abstract*.

Bianchi-Hall, C., Carter, T.E., Bailey, M., Mian, M., Rufty, T., Ashley, D., Boerma, H., Arellano, C., Hussey, R., and Parrott, W. (2000). Aluminum tolerance associated with quantitative trait loci derived from soybean PI 416937 in hydroponics. *Crop Sci.* 40**,** 538-545.

Carpentieri-Pipolo, V., Pipolo, A., Abdel-Haleem, H., Boerma, H., and Sinclair, T. (2012). Identification of QTLs associated with limited leaf hydraulic conductance in soybean. *Euphytica* 186**,** 679-686.

Chen, H., Cui, S., Fu, S., Gai, J., and Yu, D. (2008). Identification of quantitative trait loci associated with salt tolerance during seedling growth in soybean (Glycine max L.). *Crop Pasture Sci.* 59**,** 1086-1091.

Cheng, J., Yuan, C., and Graham, T.L. (2011). Potential defense-related prenylated isoflavones in lactofen-induced soybean. *Phytochemistry* 72**,** 875-881.

Choi, J.N., Kim, J., Lee, M.Y., Park, D.K., Hong, Y.-S., and Lee, C.H. (2010). Metabolomics revealed novel isoflavones and optimal cultivation time of Cordyceps militaris fermentation. *J. Agric.Food Chem.* 58**,** 4258-4267.

Clarke, J.D., Alexander, D.C., Ward, D.P., Ryals, J.A., Mitchell, M.W., Wulff, J.E., and Guo, L. (2013). Assessment of Genetically Modified Soybean in Relation to Natural Variation in the Soybean Seed Metabolome. *Scientific Rep.* 3. doi:10.1038/srep03082.

Collakova, E., Aghamirzaie, D., Fang, Y., Klumas, C., Tabataba, F., Kakumanu, A., Myers, E., Heath, L.S., and Grene, R. (2013). Metabolic and Transcriptional Reprogramming in Developing Soybean (Glycine max) Embryos. *Metabolites* 3**,** 347-372.

Cornelious, B., Chen, P., Chen, Y., De Leon, N., Shannon, J., and Wang, D. (2005). Identification of QTLs underlying water-logging tolerance in soybean. *Mol. Breed.* 16**,** 103-112.

Crossa, J., De Los Campos, G., Pérez, P., Gianola, D., Burgueño, J., Araus, J.L., Makumbi, D., Singh, R.P., Dreisigacker, S., and Yan, J. (2010). Prediction of genetic values of quantitative traits in plant breeding using pedigree and molecular markers. *Genetics* 186**,** 713-724.

Crossa, J., Pérez, P., De Los Campos, G., Mahuku, G., Dreisigacker, S., and Magorokosho, C. (2011). Genomic selection and prediction in plant breeding. *J. Crop Improv.* 25**,** 239-261.

Cui, D., Zhang, Q., Li, M., Hartman, G.L., and Zhao, Y. (2010). Image processing methods for quantitatively detecting soybean rust from multispectral images. *Biosyst. Eng.* 107**,** 186-193.

Du, W., Wang, M., Fu, S., and Yu, D. (2009a). Mapping QTLs for seed yield and drought susceptibility index in soybean (*Glycine max* L.) across different environments. *J. Genet. Genomics* 36**,** 721-731.

Du, W., Yu, D., and Fu, S. (2009b). Detection of quantitative trait loci for yield and drought tolerance traits in soybean using a recombinant inbred line population. *J. Integr. Plant Biol.* 51**,** 868-878.

Fingerová, H., and Koplík, R. (1999). Study of minerals and trace element species in soybean flour. *Fresenius' J. Anal. Chem.* 363**,** 545-549.

Galant, A., Koester, R.P., Ainsworth, E.A., Hicks, L.M., and Jez, J.M. (2012). From climate change to molecular response: redox proteomics of ozone‐induced responses in soybean. *New Phytol.* 194**,** 220-229.

Gouy, M., Rousselle, Y., Bastianelli, D., Lecomte, P., Bonnal, L., Roques, D., Efile, J.-C., Rocher, S., Daugrois, J., and Toubi, L. (2013). Experimental assessment of the accuracy of genomic selection in sugarcane. *Theor. Appl. Genet.* 126**,** 2575-2586.

Grattapaglia, D., and Resende, M.D. (2011). Genomic selection in forest tree breeding. *Tree Genet. Genomes* 7**,** 241-255.

Grattapaglia, D., Vaillancourt, R.E., Shepherd, M., Thumma, B.R., Foley, W., Külheim, C., Potts, B.M., and Myburg, A.A. (2012). Progress in Myrtaceae genetics and genomics: Eucalyptus as the pivotal genus. *Tree Genet. Genomes* 8**,** 463-508.

Guzman, P., Diers, B., Neece, D., St Martin, S., Leroy, A., Grau, C., Hughes, T., and Nelson, R. (2007). QTL associated with yield in three backcross-derived populations of soybean. *Crop Sci.* 47**,** 111-122.

Ha, B.-K., Vuong, T.D., Velusamy, V., Nguyen, H.T., Shannon, J.G., and Lee, J.-D. (2013). Genetic mapping of quantitative trait loci conditioning salt tolerance in wild soybean (Glycine soja) PI 483463. *Euphytica***,** 1-10.

Hajduch, M., Ganapathy, A., Stein, J.W., and Thelen, J.J. (2005). A systematic proteomic study of seed filling in soybean. Establishment of high-resolution two-dimensional reference maps, expression profiles, and an interactive proteome database. *Plant Physiol.* 137**,** 1397-1419.

Hamwieh, A., Tuyen, D., Cong, H., Benitez, E., Takahashi, R., and Xu, D. (2011). Identification and validation of a major QTL for salt tolerance in soybean. *Euphytica* 179**,** 451-459.

Hamwieh, A., and Xu, D. (2008). Conserved salt tolerance quantitative trait locus (QTL) in wild and cultivated soybeans. *Breed. Sci.* 58**,** 355-359.

Han, C., Yin, X., He, D., and Yang, P. (2013). Analysis of Proteome Profile in Germinating Soybean Seed, and Its Comparison with Rice Showing the Styles of Reserves Mobilization in Different Crops. *PloS one* 8**,** e56947.

Hao, D., Chao, M., Yin, Z., and Yu, D. (2012a). Genome-wide association analysis detecting significant single nucleotide polymorphisms for chlorophyll and chlorophyll fluorescence parameters in soybean (Glycine max) landraces. *Euphytica* 186**,** 919-931.

Hao, D., Cheng, H., Yin, Z., Cui, S., Zhang, D., Wang, H., and Yu, D. (2012b). Identification of single nucleotide polymorphisms and haplotypes associated with yield and yield components in soybean (Glycine max) landraces across multiple environments. *Theor. Appl. Genet.* 124**,** 447-458.

Heffner, E.L., Jannink, J.-L., and Sorrells, M.E. (2011). Genomic selection accuracy using multifamily prediction models in a wheat breeding program. *Plant Genome* 4**,** 65-75.

Hofheinz, N., Borchardt, D., Weissleder, K., and Frisch, M. (2012). Genome-based prediction of test cross performance in two subsequent breeding cycles. *Theor. Appl. Genet.* 125**,** 1639-1645.

Hossain, Z., Hajika, M., and Komatsu, S. (2012). Comparative proteome analysis of high and low cadmium accumulating soybeans under cadmium stress. *Amino Acids* 43**,** 2393-2416.

Hu, Z., Li, Y., Song, X., Han, Y., Cai, X., Xu, S., and Li, W. (2011). Genomic value prediction for quantitative traits under the epistatic model. *BMC Genetics* 12**,** 15.

Hwang, E.-Y., Song, Q., Jia, G., Specht, J.E., Hyten, D.L., Costa, J., and Cregan, P.B. (2014). A genome-wide association study of seed protein and oil content in soybean. *BMC Genomics* 15**,** 1.

Isik, F., Whetten, R., Zapata-Valenzuela, J., Ogut, F., and Mckeand, S. (2011). "Genomic selection in loblolly pine-from lab to field", in: *BMC Proceedings*: BioMed Central Ltd), I8.

Ito, S., Suzui, N., Kawachi, N., Ishii, S., Ishioka, N.S., and Fujimaki, S. (2010). Real-time analysis of translocation of photosynthates to nodules in soybean plants using ^11^CO_2_ with a positron-emitting tracer imaging system (PETIS). *Radioisotopes (Tokyo)* 59**,** 145-154.

Iwata, H., Hayashi, T., Terakami, S., Takada, N., Sawamura, Y., and Yamamoto, T. (2013). Potential assessment of genome-wide association study and genomic selection in Japanese pear Pyrus pyrifolia. *Breed. Sci.* 63**,** 125.

Jarquín, D., Crossa, J., Lacaze, X., Du Cheyron, P., Daucourt, J., Lorgeou, J., Piraux, F., Guerreiro, L., Pérez, P., and Calus, M. (2013). A reaction norm model for genomic selection using high-dimensional genomic and environmental data. *Theor. Appl. Genet.***,** 1-13.

Jia, Y., and Jannink, J.-L. (2012). Multiple-trait genomic selection methods increase genetic value prediction accuracy. *Genetics* 192**,** 1513-1522.

Kassem, M., Shultz, J., Meksem, K., Cho, Y., Wood, A., Iqbal, M., and Lightfoot, D. (2006). An updated ‘Essex’by ‘Forrest’linkage map and first composite interval map of QTL underlying six soybean traits. *Theor. Appl. Genet.* 113**,** 1015-1026.

Kassem, M.A., Meksem, K., Kang, C., Njiti, V., Kilo, V., Wood, A., and Lightfoot, D. (2004). Loci underlying resistance to manganese toxicity mapped in a soybean recombinant inbred line population of ‘Essex2019; x ‘Forrest’. *Plant Soil* 260**,** 197-204.

King, K.E., Peiffer, G.A., Reddy, M., Lauter, N., Lin, S.F., Cianzio, S., and Shoemaker, R.C. (2013). Mapping of Iron and Zinc Quantitative Trait Loci in Soybean for Association to Iron Deficiency Chlorosis Resistance. *J. Plant Nutrit*.36, 2132-2153.

Ko, B.-K., Kim, K.M., Hong, Y.-S., and Lee, C.-H. (2010). Metabolomic Assessment of Fermentative Capability of Soybean Starter Treated with High Pressure. *J. Agr. Food Chem.* 58**,** 8738-8747.

Komatsu, S., Makino, T., and Yasue, H. (2013). Proteomic and Biochemical Analyses of the Cotyledon and Root of Flooding-Stressed Soybean Plants. *PLoS* o*ne* 8**,** e65301.

Komatsu, S., Yamamoto, R., Nanjo, Y., Mikami, Y., Yunokawa, H., and Sakata, K. (2009). A comprehensive analysis of the soybean genes and proteins expressed under flooding stress using transcriptome and proteome techniques. *J. Proteome Res.* 8**,** 4766-4778.

Koplı́K, R., Pavelková, H., Cincibuchová, J., Mestek, O., Kvasnička, F., and Suchánek, M. (2002). Fractionation of phosphorus and trace elements species in soybean flour and common white bean seeds by size exclusion chromatography–inductively coupled plasma mass spectrometry. *J. Chromatography B* 770**,** 261-273.

Korir, P.C., Qi, B., Wang, Y., Zhao, T., Yu, D., Chen, S., and Gai, J. (2011). A study on relative importance of additive, epistasis and unmapped QTL for aluminium tolerance at seedling stage in soybean. *Plant Breed.* 130**,** 551-562.

Korir, P.C., Zhang, J., Wu, K., Zhao, T., and Gai, J. (2013). Association mapping combined with linkage analysis for aluminum tolerance among soybean cultivars released in Yellow and Changjiang River Valleys in China. *Theor. Appl. Genet.* 126, 1659-75.

Lee, G., Boerma, H., Villagarcia, M., Zhou, X., Carter Jr, T., Li, Z., and Gibbs, M. (2004). A major QTL conditioning salt tolerance in S-100 soybean and descendent cultivars. *Theor. Appl. Genet.* 109**,** 1610-1619.

Li, D., Pfeiffer, T., and Cornelius, P. (2008). Soybean QTL for Yield and Yield Components Associated with Alleles. *Crop Sci.* 48**,** 571-581.

Li, Y., Wang, Y., Tong, Y., Gao, J., Zhang, J., and Chen, S. (2005). QTL mapping of phosphorus deficiency tolerance in soybean (Glycine max L. Merr.). *Euphytica* 142**,** 137-142.

Li, Y., Smulders, M., Chang, R., and Qiu, L.. (2011). Genetic diversity and association mapping in a collection of selected Chinese soybean accessions based on SSR marker analysis. *Conserv. Genet.* 12**,** 1145-1157.

Lorenzana, R.E., and Bernardo, R. (2009). Accuracy of genotypic value predictions for marker-based selection in biparental plant populations. *Theor. Appl. Genet.* 120**,** 151-161.

Lu, Y., Lam, H., Pi, E., Zhan, Q., Tsai, S., Wang, C., Kwan, Y., and Ngai, S. (2013). Comparative Metabolomics in Glycine max and Glycine soja under Salt Stress To Reveal the Phenotypes of Their Offspring. *J.Agr. Food Chem.* 61**,** 8711-8721.

Mamidi, S., Chikara, S., Goos, R.J., Hyten, D.L., Annam, D., Moghaddam, S.M., Lee, R.K., Cregan, P.B., and Mcclean, P.E. (2011). Genome-wide association analysis identifies candidate genes associated with iron deficiency chlorosis in soybean. *Plant Genome* 4**,** 154-164.

Mataveli, L.R.V., Fioramonte, M., Gozzo, F.C., and Arruda, M.a.Z. (2012). Improving metallomics information related to transgenic and non-transgenic soybean seeds using 2D-HPLC-ICP-MS and ESI-MS/MS. *Metallomics* 4**,** 373-378.

Mataveli, L.R.V., Pohl, P., Mounicou, S., Arruda, M.a.Z., and Szpunar, J. (2010). A comparative study of element concentrations and binding in transgenic and non-transgenic soybean seeds. *Metallomics* 2**,** 800-805.

Matsuura, H., Itoh, H., Tomoda, S., Shiraishi, N., and Uno, Y. (2010). Identification of signaling transmitter to activate nitrate reductase in vegetable leaves by near-infrared hyperspectral imaging system. *Agricontrol*, 160-165.

Mestek, O., Komı́Nková, J., Koplı́K, R., Borková, M., and Suchánek, M. (2002). Quantification of copper and zinc species fractions in legume seeds extracts by SEC/ICP-MS: validation and uncertainty estimation. *Talanta* 57**,** 1133-1142.

Mian, M., Ashley, D., and Boerma, H. (1998). An additional QTL for water use efficiency in soybean. *Crop Sci.* 38**,** 390-393.

Mian, M., Bailey, M., Ashley, D., Wells, R., Carter, T., Parrott, W., and Boerma, H. (1996). Molecular markers associated with water use efficiency and leaf ash in soybean. *Crop Sci.* 36**,** 1252-1257.

Molnar, S.J., Charette, M., and Cober, E.R. (2012). Mapping quantitative trait loci for water uptake in a recombinant inbred line population of natto soybean. *Can. J. Plant Sci.* 92**,** 257-266.

Mooney, B.P., and Thelen, J.J. (2004). High-throughput peptide mass fingerprinting of soybean seed proteins: automated workflow and utility of UniGene expressed sequence tag databases for protein identification. *Phytochemistry* 65**,** 1733-1744.

Natarajan, S.S., Xu, C., Bae, H., Caperna, T.J., and Garrett, W.M. (2006). Characterization of storage proteins in wild (Glycine soja) and cultivated (Glycine max) soybean seeds using proteomic analysis. *J. Agr. Food Chem.* 54**,** 3114-3120.

Niu, Y., Xu, Y., Liu, X.-F., Yang, S.-X., Wei, S.-P., Xie, F.-T., and Zhang, Y.-M. (2013). Association mapping for seed size and shape traits in soybean cultivars. *Mol. Breed.***,** 1-10.

O'shaughnessy, S., Evett, S., Colaizzi, P., and Howell, T. (2011). Using radiation thermography and thermometry to evaluate crop water stress in soybean and cotton. *Agric. Water Manag.* 98**,** 1523-1535.

Panter, S., Thomson, R., De Bruxelles, G., Laver, D., Trevaskis, B., and Udvardi, M. (2000). Identification with proteomics of novel proteins associated with the peribacteroid membrane of soybean root nodules. *Mol. Plant-Microbe Interact.* 13**,** 325-333.

Pérez-Rodríguez, P., Gianola, D., González-Camacho, J.M., Crossa, J., Manès, Y., and Dreisigacker, S. (2012). Comparison Between Linear and Non-parametric Regression Models for Genome-Enabled Prediction in Wheat. *Genes Genomes Genet.* 2**,** 1595-1605.

Qin, J., Gu, F., Liu, D., Yin, C., Zhao, S., Chen, H., Zhang, J., Yang, C., Zhan, X., and Zhang, M. (2013). Proteomic analysis of elite soybean Jidou17 and its parents using iTRAQ-based quantitative approaches. *Proteome Sci.* 11**,** 12.

Resende, M., Del Valle, P.M., Acosta, J., Resende, M., Grattapaglia, D., and Kirst, M. (2011). "Stability of Genomic Selection prediction models across ages and environments", in: *BMC Proceedings*: BioMed Central Ltd), O14.

Resende, M., Munoz, P., Acosta, J., Peter, G., Davis, J., Grattapaglia, D., Resende, M., and Kirst, M. (2012a). Accelerating the domestication of trees using genomic selection: accuracy of prediction models across ages and environments. *New Phytol.* 193**,** 617-624.

Resende, M.F., Muñoz, P., Resende, M.D., Garrick, D.J., Fernando, R.L., Davis, J.M., Jokela, E.J., Martin, T.A., Peter, G.F., and Kirst, M. (2012b). Accuracy of genomic selection methods in a standard data set of loblolly pine (Pinus taeda L.). *Genetics* 190**,** 1503-1510.

Ribeiro, A.S., Gouveia, L.R., Barros, C.J.P., Firmino, P.R.A., and Silva, R.O. (2014). Discriminating gamma-irradiated soybean seeds by ^1^H NMR-based metabonomics. *Food Control* 36**,** 266-272.

Riedelsheimer, C., Technow, F., and Melchinger, A.E. (2012). Comparison of whole-genome prediction models for traits with contrasting genetic architecture in a diversity panel of maize inbred lines. *BMC Genomics* 13**,** 452.

Rong, Z., Xianzhi, W., Haifeng, C., Xiaojuan, Z., Zhihui, S., Xuejun, W., Shuping, C., Dezhen, Q., Xinan, Z., and Jiangsheng, W. (2009). QTL analysis of lodging and related traits in soybean. *Acta Agronomica Sinica* 35**,** 57-65.

Schulz-Streeck, T., Ogutu, J.O., and Piepho, H.-P. (2013). Comparisons of single-stage and two-stage approaches to genomic selection. *Theor. Appl. Genet.* 126**,** 69-82.

Sharma, A.D., Sharma, H., and Lightfoot, D.A. (2011). The genetic control of tolerance to aluminum toxicity in the ‘Essex’by ‘Forrest’recombinant inbred line population. *Theor. Appl. Genet.* 122**,** 687-694.

Shu, Y., Yu, D., Wang, D., Bai, X., Zhu, Y., and Guo, C. (2012). Genomic selection of seed weight based on low-density SCAR markers in soybean. *Genet. Mol. Res.* 12**,** 2178-2188.

Sobhanian, H., Razavizadeh, R., Nanjo, Y., Ehsanpour, A.A., Jazii, F.R., Motamed, N., and Komatsu, S. (2010). Proteome analysis of soybean leaves, hypocotyls and roots under salt stress. *Proteome Sci.* 8**,** 19.

Swigonska, S., and Weidner, S. (2013). Proteomic analysis of response to long-term continuous stress in roots of germinating soybean seeds. *J. Plant Physiol.*. 170, 470-479.

Tuyen, D., Zhang, H., and Xu, D. (2013). Validation and high-resolution mapping of a major quantitative trait locus for alkaline salt tolerance in soybean using residual heterozygous line. *Mol. Breed.* 31**,** 79-86.

Vollmann, J., Walter, H., Sato, T., and Schweiger, P. (2011). Digital image analysis and chlorophyll metering for phenotyping the effects of nodulation in soybean. *Computer. Electronics Agric.* 75**,** 190-195.

Wan, J., Liu, W., Li, C., and Zhang, W.-Q. (2010). Determination of Mineral Elements in Soybean from Different Producing Areas by ICP-AES. *Spectros. Spect. Anal.* 30**,** 543-545.

Wan, J., Torres, M., Ganapathy, A., Thelen, J., Dague, B.B., Mooney, B., Xu, D., and Stacey, G. (2005). Proteomic analysis of soybean root hairs after infection by Bradyrhizobium japonicum. *Mol. Plant-Microbe Interact.* 18**,** 458-467.

Wang, J., Mcclean, P.E., Lee, R., Goos, R.J., and Helms, T. (2008). Association mapping of iron deficiency chlorosis loci in soybean (Glycine max L. Merr.) advanced breeding lines. *Theor. Appl. Genet.* 116**,** 777-787.

Wel, Z., Shen, L., Rui, Y., and Jiao, C. (2008). Application of ICP-MS to the detection of 22 elements in transgenic soybean oil. *Guang pu xue yu guang pu fen xi= Guang pu* 28**,** 1398-1399.

Wu, W., Zhang, Q., Zhu, Y., Lam, H.-M., Cai, Z., and Guo, D. (2008). Comparative metabolic profiling reveals secondary metabolites correlated with soybean salt tolerance. *J. Agr. Food Chem.* 56**,** 11132-11138.

Würschum, T., Abel, S., and Zhao, Y. (2014). Potential of genomic selection in rapeseed (Brassica napus L.) breeding. *Plant Breed.* 1, 45-51.

Würschum, T., Reif, J.C., Kraft, T., Janssen, G., and Zhao, Y. (2013). Genomic selection in sugar beet breeding populations. *BMC Genet.* 14**,** 85.

Xu, C., Sullivan, J.H., Garrett, W.M., Caperna, T.J., and Natarajan, S. (2008). Impact of solar ultraviolet-B on the proteome in soybean lines differing in flavonoid contents. *Phytochemistry* 69**,** 38-48.

Xu, X.-Y., Fan, R., Zheng, R., Li, C.-M., and Yu, D.-Y. (2011). Proteomic analysis of seed germination under salt stress in soybeans. *J. Zhejiang Univ. Sci. B.* 12**,** 507-517.

Yamaguchi, M., Valliyodan, B., Zhang, J., Lenoble, M.E., Yu, O., Rogers, E.E., Nguyen, H.T., and Sharp, R.E. (2010). Regulation of growth response to water stress in the soybean primary root. I. Proteomic analysis reveals region‐specific regulation of phenylpropanoid metabolism and control of free iron in the elongation zone. *Plant Cell Environ.*33**,** 223-243.

Yan, P.-M., Wang, W.-Y., Rui, Y.-K., Zhang, F.-S., and Jin, Y.-H. (2007). Application of ICP-MS/ICP-AES to the Detection of Wholesome Elements and Heavy Metals in Soybean from Northeastern China. *Spectro. Spectral. Anal.* 27**,** 1629.

Zhang, W.B., Qiu, P.C., Jiang, H.W., Liu, C.Y., Li, C.D., Hu, G.H., and Chen, Q.S. (2012). Dissection of genetic overlap of drought and low-temperature tolerance QTLs at the germination stage using backcross introgression lines in soybean. *Mol. Biol. Rep.* 39**,** 6087-6094.

Zhao, J., Zhang, Y., Bian, X., Lei, J., Sun, J., Guo, N., Gai, J., and Xing, H. (2013). A comparative proteomics analysis of soybean leaves under biotic and abiotic treatments. *Mol. Biol. Rep.* 40**,** 1553-1562.

Zhen, Y., Qi, J.L., Wang, S.S., Su, J., Xu, G.H., Zhang, M.S., Miao, L., Peng, X.X., Tian, D., and Yang, Y.H. (2007). Comparative proteome analysis of differentially expressed proteins induced by Al toxicity in soybean. *Physiologia Plantarum* 131**,** 542-554.

Zhu, D., Li, Y., Wang, D., Wu, Q., Zhang, D., and Wang, C. (2012). The Identification of Single Soybean Seed Variety by Laser Light Backscattering Imaging. *Sensor Lett.* 10**,** 1-2.

Ziegler, G., Terauchi, A., Becker, A., Armstrong, P., Hudson, K., and Baxter, I. (2013). Ionomic Screening of Field-Grown Soybean Identifies Mutants with Altered Seed Elemental Composition. *Plant Genome* 6**,** 9. doi: doi:10.3835/plantgenome2012.07.0012.

Zuo, Q.-M., Wen, Z.-X., Zhang, S.-Y., Hou, J.-F., Gai, J.-Y., Yu, D.-Y., and Xing, H. (2013). QTL Identification of the Insensitive Response to Photoperiod and Temperature in Soybean by Association Mapping. *J. Integ. Agric.* 12**,** 1423-1430.
